# Introduction to research topic: attention and consciousness in different senses

**DOI:** 10.3389/fpsyg.2013.00249

**Published:** 2013-05-01

**Authors:** Naotsugu Tsuchiya, Jeroen van Boxtel

**Affiliations:** ^1^Faculty of Medicine, Nursing and Health Sciences, School of Psychology and Psychiatry, Monash UniversityMelbourne, VIC, Australia; ^2^Japan Science and Technology AgencyTokyo, Japan; ^3^Department of Psychology, University of California Los AngelesLos Angeles, CA, USA; ^4^Division of Biology, California Institute of TechnologyPasadena, CA, USA

The question of the origin of consciousness has engaged scientists and philosophers for centuries. Early scholars relied on introspection, leading some to conclude that attention is necessary for consciousness, and in some cases equating attention and consciousness. Such a tight relationship between attention and consciousness has also been proposed by many modern theorists (Posner, 1994; Merikle and Joordens, 1997; Mack and Rock, 1998; Chun and Wolfe, 2000; O'Regan and Noe, 2001; Mole, 2008; De Brigard and Prinz, 2010; Prinz, 2011; Cohen et al., 2012). The relationship between attention and consciousness has come under increasing scrutiny with the development of neuroscientific methods. In modern neuroscience, the effects of attention are often objectively defined and measured as reduced reaction time and improved performance. Similarly, conscious awareness of an object is established by a subjective report in combination with objective forced-choice performance (Seth et al., 2008; Sandberg et al., 2011). With these measures in place, a variety of methods has been used to manipulate attention (e.g., cueing, divided attention, etc.) and consciousness [e.g., masking, crowding, and binocular rivalry (Kim and Blake, 2005)]. These empirical studies have culminated in recent proposals that attention and consciousness are supported by different neuronal processes and they are not necessarily correlated all the time (Iwasaki, 1993; Baars, 1997; Hardcastle, 1997; Kentridge et al., 1999; Naccache et al., 2002; Lamme, 2003; Woodman and Luck, 2003; Bachmann, 2006; Koch and Tsuchiya, 2007; van Boxtel et al., 2010).

Our original motivation to edit this Research Topic was threefold: (1) to gather and collect current, diverse views on the relationship between consciousness and attention, (2) to invite reviews on consciousness and attention in non-vision modalities, (3) and to invite empirical studies of consciousness and attention. As summarized below, our goals are largely achieved thanks to 17 contributions to this issue.

## Current perspectives on the relationship between consciousness and attention

Posner (2012) sets the stage for the discussion by distinguishing different ways in which “consciousness” and “attention” are used colloquially. Clarifying the three senses of consciousness, namely, the level or state of consciousness (as in coma, sleep, or awake), sensory awareness (or contents of consciousness), and voluntary control, Posner claims that the neuronal mechanisms that support each type of consciousness overlap with those for a distinct type of attention: alerting, orienting, and executive attention, respectively. Chennu and Bekinschtein (2012) investigate the workings of attention at different levels or states of consciousness. Reviewing the mismatch negativity in the auditory oddball paradigm, they survey evidence for dissociations and parallels between bottom-up and top-down attention and the level of consciousness. Marchetti (2012) largely agrees with Posner (2012), emphasizing the variety in types of attention and consciousness. By considering each type, Marchetti argues that consciousness is always associated with some kind of attention and attention is always associated with some kind of conscious perception. Chica and Bartolomeo (2012) dissect attention into endogenous/top-down and exogenous/bottom-up components, considering their relation with consciousness. They claim that endogenous attention is neither necessary nor sufficient for consciousness agreeing with some views (e.g., Koch and Tsuchiya, 2007; van Boxtel et al., 2010) while exogenous attention *is* necessary (but not sufficient) for consciousness (also see Hsu et al., 2011). They note the prefrontal parietal network (PPN) as central for both exogenous spatial attention and conscious perception. The importance of the PPN is also stressed by Bor and Seth (2012), who review recent empirical studies for identifying potential neuronal correlates of consciousness (NCCs). Based on the fact that the PPN has been commonly identified as the neuronal correlate for both attention and consciousness (Rees and Lavie, 2001), they suggest that attention is an important and necessary aspect of consciousness. As the PPN is also associated with working memory, executive control, and chunking, they argue that these cognitive functions, including attention, make up the core psychological components of consciousness. A contrasting view on the role of the PPN is provided by Tallon-Baudry (2011), who argues against the tight relationship between consciousness and attention. She raises several issues about the interpretation of previous results with respect to the PPN. For example, many previous experiments did not independently manipulate both attention and consciousness. To explain recent neural findings pointing to a dissociation of attention and consciousness, she proposes “a cumulative influence model,” where both attention and consciousness contribute to the final stage of decision making through independent paths.

Two novel theoretical ideas are put forward in this Research Topic. Hohwy (2012) proposes a framework based on predictive coding (Rao, 2005; Friston, 2009), where attention optimizes expectations about perceptual precisions, while conscious perception is a result of the prediction error minimization. Bachmann (2011) proposes to broaden the research view from focusing on modality-specific and feature-specific effects of attention to inter-modal effects of attention on consciousness. He concludes that attention and consciousness are separable and that consciousness can come about without selective attention. As Bachmann points out, the articles introduced so far build their theories mostly on the experimental evidence obtained in the visual modality. Next we will overview the articles looking outside the visual modality.

## Consciousness and attention in non-vision fields

Snyder et al. (2012) review recent experiments in auditory neuroscience, which investigated how conscious auditory perception is influenced by various high-level factors, including attention. They also review a variety of methods, including multistable stimuli and masking phenomena in the auditory domain, which will allow future research to shed a new light on the overlap in the neuronal mechanisms of attention and consciousness in audition and vision.

Keller (2011) explores the potential law-like relation between attention and consciousness in olfaction. As olfaction is very different from vision in several aspects (e.g., lack of spatial specificity, no object as a unit for selection, etc.), the concept of attention is clarified in comparison with that in vision. Then, Keller goes on to speculate about possible neuronal loci for attentional selection and conscious processing for olfaction. He concludes that attention is necessary for olfactory consciousness.

Lou et al. (2011) and De Brigard (2012) extend the discussion into non-sensory modalities. Lou et al. (2011) examine the role of the brain regions that locate around the midline, (i.e., paralimbic, resting-state, or default-mode network) in self-awareness and self-control, concluding that the network integrates attention, awareness and emotion to allocate brain resources. Looking at memory research, De Brigard (2012) dissect the kinds of attention important for memories. De Brigard follows the recent proposal for a distinction between internal and external attention (Chun et al., 2011) and argues that internal attention is necessary, but probably not sufficient, for conscious retrieval of memories.

## Empirical studies on attention and consciousness

The Research Topic concludes with several empirical studies. While top-down selective attention is commonly assumed to be required to bind features into objects, Rosenholtz et al. (2012) argue that recent changes in the understanding of peripheral vision provides an alternative view. Their texture tiling model [also see perceptual metamers (Freeman and Simoncelli, 2011)] successfully explains why complex tasks, such as pop-out in visual search and natural scene categorization (Li et al., 2002), can be performed well in the periphery where the accuracy of information is severely impaired. Their model does not resort to top-down attention in accomplishing the complex visual tasks. Moutoussis (2012) reviews the findings on perceptual timing and binding of visual features, concluding that misbinding illusions are due to differences in neural processing times as well as exogenous attention. Perry and Fallah (2012) conducted a psychophysics study to investigate the influence of bottom-up and top-down attention on a visual illusion called “motion direction repulsion.” They found that attentional manipulation via color affects processing speed without changing the conscious perception of motion. Delevoye-Turrell and Bobineau (2012) investigated the effects of lowered and heightened attention on motor consciousness using a metacognitive approach (e.g., reproduction of a motion trajectory) for reflex-like stimulus-based and deliberate intention-based actions. Reproduction quality depended on how skillful subjects were in meditation, presumably reflecting the effectiveness of attentional control on the body. Finally, Willenbockel et al. (2012) recorded intracranial neuronal activity from the insula and amygdala of awake human patients to characterize the effects of visibility using continuous flash suppression (Tsuchiya and Koch, 2005) as well as using spatial-frequency “bubbles” (Willenbockel et al., 2010). They found that low spatial frequency information of invisible faces distinctively activated these regions and that activation by invisible faces precedes those evoked by visible faces.

### Questions for future studies

Over all the articles collected here, the most recurring issue is whether attention is necessary for conscious perception (Bachmann, 2011; Keller, 2011; Tallon-Baudry, 2011; Bor and Seth, 2012; Chennu and Bekinschtein, 2012; Chica and Bartolomeo, 2012; De Brigard, 2012; Marchetti, 2012; Posner, 2012; Rosenholtz et al., 2012). There are two aspects in this debate.

First, the role of the PPN for attention and consciousness is disputed (Tallon-Baudry, 2011; Bor and Seth, 2012; Chica and Bartolomeo, 2012). While meta-analyses of studies on consciousness and (exogenous) attention (Bor and Seth, 2012; Chica and Bartolomeo, 2012) point to a large overlap in the PPN for both attention and consciousness, Tallon-Baudry (2011) argues that not all the experiments have shown consciousness-related activation in the PPN (e.g., Tse et al., 2005), that some of the PPN activation may be related to a confound related to report or resolution of conflict (Knapen et al., 2011; van Boxtel and Tsuchiya, 2013), and that most studies did not independently manipulate attention and consciousness. These are all empirical issues, which can be relatively easy to address in the future studies.

The second issue is a bit trickier. Some claim that attention is always necessary, not only for vision but also for olfaction (Keller, 2011) and memory (De Brigard, 2012). Such an argument could be countered by everyday examples, such as peripheral vision (Rosenholtz et al., 2012), unexpected strong olfactory stimuli (Keller, 2011), and the feeling of familiarity (De Brigard, 2012), all of which appear to give rise to conscious experience without deliberate attentional amplification. This view is also supported by conscious perception of an isolated stimulus, because top-down attention can function when only the sensory inputs are competing with each other (Koch and Tsuchiya, 2007; van Boxtel et al., 2010). However, it is possible to argue that even these cases require some amount of attention, because attention is present everywhere in some diffuse way (De Brigard and Prinz, 2010; Keller, 2011; Cohen et al., 2012; Marchetti, 2012).

Currently, no psychophysical experimentation in humans can prove or disprove such a hypothesis, as it seems impossible to induce the state of no attention in humans. However, the advent of optogenetics in animal research might allow us to create a situation where no attentional activation, fed back from the PPN, arrives at the visual cortex (Tsuchiya et al., 2012). It remains to be seen if such animals without (top-down) attention can consciously see an isolated object or not.

Even if it turns out animals can visually perceive an isolated object without top-down attention, it remains unclear if inter-modal attention is required for conscious perception in a non-dominant modality, such as olfaction and memory. It is plausible that regardless of the inputs, there may be always competition between modalities (e.g., dominant vision vs. non-dominant olfaction) and between times (e.g., dominant present vs. non-dominant past memory or future planning). By default, the dominant modality may be a winning coalition (i.e., present visual input) requiring nearly no top-down attentional amplification to be consciously experienced while non-dominant modalities might require some level of attentional amplification to reach consciousness.

We hope these articles will inspire the readers for further conceptual and empirical work on the issue of the relationship between consciousness and attention. Untangling this relation is the necessary step toward uncovering the neuronal basis of consciousness.
